# Missing for partnership: understanding nucleosomal de novo DNA cytosine methylation by a spliced DNMT3 complex

**DOI:** 10.1038/s41392-021-00461-2

**Published:** 2021-01-29

**Authors:** Min Zhang, Haitao Li

**Affiliations:** MOE Key Laboratory of Protein Sciences, Beijing Advanced Innovation Center for Structural Biology, Tsinghua-Peking Center for Life Sciences, School of Medicine, Tsinghua University, Beijing, China

**Keywords:** Structural biology, Epigenetics

In a recent paper published in Nature, Xu et al. present a cryo-EM structure of a ‘spliced’ de novo DNA methyltransferase complex, DNMT3A2-DNMT3B3, bound to a mono-nucleosome with extended linker DNA.^1^ Reinforced by biochemical assays and genomic studies, this structure reveals the molecular mechanism underlying de novo cytosine methylation on nucleosomal substrate. Notably, the catalytically inactive DNMT3B3 occupies the nucleosome core and orients the active DNMT3A2 to target the linker DNA as its substrate, suggesting the necessity of chromosomal remodeler in the deposition of DNA methylation on nucleosomes.

5-methyl-cytosine (5mC) is one of the best characterized epigenetic modification. It mainly exists at CpG dinucleotides on mammalian genome except CpG islands. Coordinated with histone modifications, 5mC plays an essential role in gene regulation, as well as chromatin-based events such as imprinting, X chromosome inactivation, and repetitive elements silencing. 5mC is established de novo by two DNMT3 enzymes, DNMT3A and DNMT3B, assisted by catalytically inactive accessory paralogues, such as DNMT3L and DNMT3B3. Previous structure studies have revealed the molecular mechanisms underlying the coordination between histone modification and DNMT3A in 5mC establishment,^2,3^ as well as substrate recognition on naked DNA;^4^ yet, it remains elusive that how DNMT3 catalyze 5mC in the context of chromatin landscape, its natural substrate, especially with the fact that DNMT3 prefers linker DNA instead of nucleosomal core DNA for methylation in vitro.

**Fig. 1:**
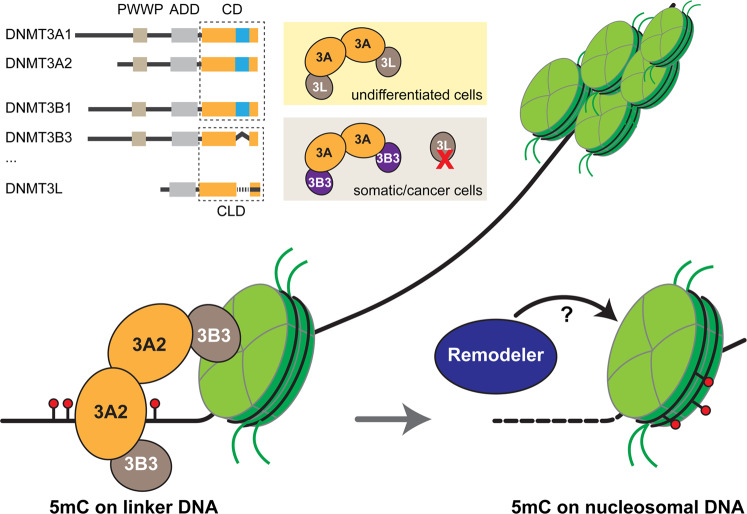
DNA methylation by a spliced DNMT3A2-3B3 complex on a single nucleosome

In this regard, Xu et al. determined the structure of DNMT3A2-DNMT3B3 in complex with mono-nucleosome flanked by 10 bp linker on both sides, the basic repeating subunits of chromatin (Fig. 1). Overall, the catalytic domain (CD) of DNMT3A2, the catalytic-like domain (CLD) of DNMT3B3, and the nucleosome, as well as its flanking DNA are well resolved in the complex; in particular, the 3B3-3A2-3A2-3B3 heterotetramer adopts a highly similar conformation as DNMT3A-DNMT3L reported before. Notably, this heterotetramer interacts asymmetrically with its substrate, with one CLD of 3B3 anchoring the acidic patch of nucleosome, which orientates the two CDs of 3A2, as well as another CLD of 3B3 away from the core nucleosome. In this way, the catalytic center of the distal 3A2 is directed to the linker DNA, with its target recognition domain (TRD) loop and the catalytic loop inserted into the major groove and the minor groove, respectively. Two key residues, R740 and R743 of 3B3, was demonstrated to play an essential role in anchoring 3B3 to the acidic patch, mutagenesis of which compromised nucleosomal binding and DNA methylation. Based on this binding pattern, Xu et al. leveraged micrococcal nuclease digestion sequencing (MNase-seq) to explore the occupancy of DNMT3A2-DNMT3B3 on linker DNA in solution. Indeed, the DNMT complex predominantly locates at about 10 bp on either side away from the nucleosome; however, one side is unexpectedly more preferred than the other, which may result from the intrinsic geometry of the complex.

Also, this structure reinforces the role of DNMT3B3, a catalytically inactive splicing isoform of DNMT3B, as an DNMT3L-like accessory factor, which is consistent with its functional substitution of DNMT3L in somatic cells.^5^ The switch of a ‘loss-of-catalytic function’ DNMT3B isoform into a ‘gain-of-function’ accessory partner emphasizes an intriguing regulatory mechanism driven by alternative splicing during de novo DNA cytosine methylation (Fig. 1).

Previous studies established the crucial regulatory role of the unmodified histone H3 tail in DNMT3A activation.^3^ In this study, Xu et al. demonstrated the nucleosomal acidic patch as another substantial element mediating the binding of DNMT3A2-DNMT3B3 to nucleosome. In addition, mutants (R740E and R743E) deficient in acidic patch binding were observed to enrich in gene body and colocalize with H3K36me3, re-emphasizing the interplay among histone H3 tail, PWWP and ADD domains in DNMT complex recruitment. Although no reliable density is identified for ADD and PWWP domains so far, this structure provides us inspiring framework to study the crosstalk between histone modification and DNA methylation on chromatin landscape in the future.

Although 5mC tends to localize at nucleosome core instead of linker DNA genome wide, DNMT3A hardly methylates DNA within the nucleosome-wrapped DNA in vitro. Here, the complex structure nicely reveals its underlying mechanism, and implies that DNMT3A methylation is coupled with remodeling events in cells to relocate 5mC to the core nucleosome (Fig. 1). Indeed, the dependence on remodelers (e.g., DDM1/Lsh) for proper 5mC deposition at nucleosome-bound DNA has been demonstrated on genome.^1^

Taken together, this study sheds light on the molecular mechanisms underlying de novo DNA methylation by presenting the complex structure of DNMT3A2-DNMT3B3 functioning on extended nucleosome for the first time. Further efforts are inspired to elucidate the crosstalk between DNA methylation, histone modification, and nucleosome remodeling by this intriguing study.
